# 3-(4-Chloro­phen­yl)-5-(4-fluoro­phen­yl)-4,5-dihydro-1*H*-pyrazole-1-carbothio­amide

**DOI:** 10.1107/S1600536813004492

**Published:** 2013-02-20

**Authors:** Bakr F. Abdel-Wahab, Hanan A. Mohamed, Seik Weng Ng, Edward R. T. Tiekink

**Affiliations:** a Applied Organic Chemistry Department, National Research Centre, Dokki, 12622 Giza, Egypt; b Department of Chemistry, Faculty of Science, Mansoura University, ET-35516 Mansoura, Egypt; c Department of Chemistry, University of Malaya, 50603 Kuala Lumpur, Malaysia; d Chemistry Department, Faculty of Science, King Abdulaziz University, PO Box 80203 Jeddah, Saudi Arabia

## Abstract

In the title compound, C_16_H_13_ClFN_3_S, the pyrazole ring adopts an envelope conformation with the methine C atom being the flap atom. The chloro- and fluoro­benzene rings are twisted out of the plane of the pyrazole ring [dihedral angles = 15.12 (11) and 80.55 (10)°, respectively]. The amine group is orientated towards a ring N atom, forming an intra­molecular N—H⋯N hydrogen bond. This H atom also forms a hydrogen bond to the F atom, which along with N—H⋯S hydrogen bonding leads to a supra­molecular chain along the *c* axis. Connections between chains of the type Cl⋯π lead to a layer in the *bc* plane.

## Related literature

For the biological activity of pyrazolin-1-yl­thia­zoles, see: Abdel-Wahab *et al.* (2009 ▶, 2012 ▶); Chimenti *et al.* (2010 ▶). For related structures, see: Chantrapromma *et al.* (2012 ▶); Abdel-Wahab *et al.* (2013 ▶).
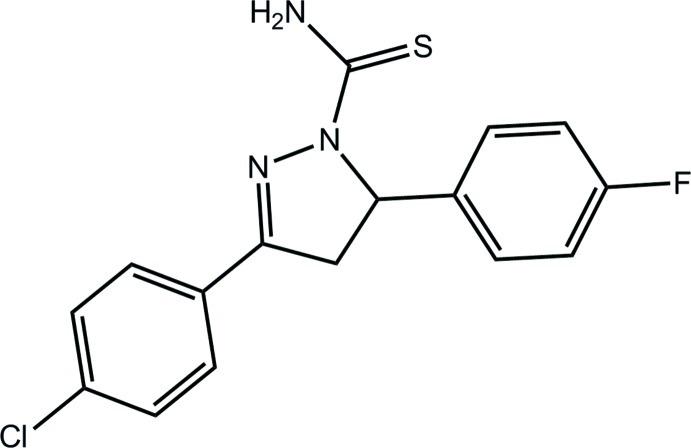


## Experimental

### 

#### Crystal data

C_16_H_13_ClFN_3_S
*M* *_r_* = 333.80
Monoclinic, 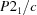

*a* = 14.5402 (9) Å
*b* = 11.2700 (8) Å
*c* = 9.5169 (6) Å
β = 103.850 (6)°
*V* = 1514.17 (17) Å^3^
*Z* = 4
Mo *K*α radiation
μ = 0.40 mm^−1^
*T* = 295 K
0.40 × 0.30 × 0.20 mm

#### Data collection

Agilent SuperNova Dual diffractometer with an Atlas detector
Absorption correction: multi-scan (*CrysAlis PRO*; Agilent, 2011 ▶) *T*_min_ = 0.898, *T*_max_ = 1.000
10191 measured reflections
3478 independent reflections
2570 reflections with *I* > 2σ(*I*)
*R*_int_ = 0.031

#### Refinement

*R*[*F* ^2^ > 2σ(*F* ^2^)] = 0.041
*wR*(*F* ^2^) = 0.111
*S* = 1.01
3478 reflections
199 parameters
H-atom parameters constrained
Δρ_max_ = 0.19 e Å^−3^
Δρ_min_ = −0.28 e Å^−3^

### 

Data collection: *CrysAlis PRO* (Agilent, 2011 ▶); cell refinement: *CrysAlis PRO*; data reduction: *CrysAlis PRO*; program(s) used to solve structure: *SHELXS97* (Sheldrick, 2008 ▶); program(s) used to refine structure: *SHELXL97* (Sheldrick, 2008 ▶); molecular graphics: *ORTEP-3 for Windows* (Farrugia, 2012 ▶) and *DIAMOND* (Brandenburg, 2006 ▶); software used to prepare material for publication: *publCIF* (Westrip, 2010 ▶).

## Supplementary Material

Crystal structure: contains datablock(s) global, I. DOI: 10.1107/S1600536813004492/hg5293sup1.cif

Structure factors: contains datablock(s) I. DOI: 10.1107/S1600536813004492/hg5293Isup2.hkl

Supplementary material file. DOI: 10.1107/S1600536813004492/hg5293Isup3.cml

Additional supplementary materials:  crystallographic information; 3D view; checkCIF report

## Figures and Tables

**Table 1:** Hydrogen-bond geometry (Å, °) *Cg*1 is the centroid of the C1–C6 ring.

*D*—H⋯*A*	*D*—H	H⋯*A*	*D*⋯*A*	*D*—H⋯*A*
N3—H31⋯N1	0.88	2.24	2.617 (2)	106
N3—H31⋯F1^i^	0.88	2.41	3.257 (2)	163
N3—H32⋯S1^ii^	0.88	2.81	3.5203 (19)	139
C4—Cl1⋯*Cg*1^iii^	1.735 (2)	3.9240 (12)	4.183 (2)	86.17 (17)

Symmetry codes: (i) 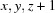; (ii) 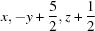; (iii) 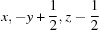.

**Fig. 1.**
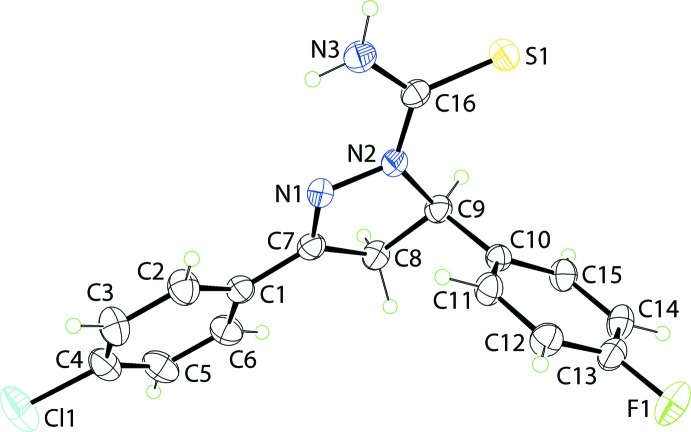
The molecular structure of (I) showing the atom-labelling scheme and displacement ellipsoids at the 35% probability level.

**Fig. 2.**
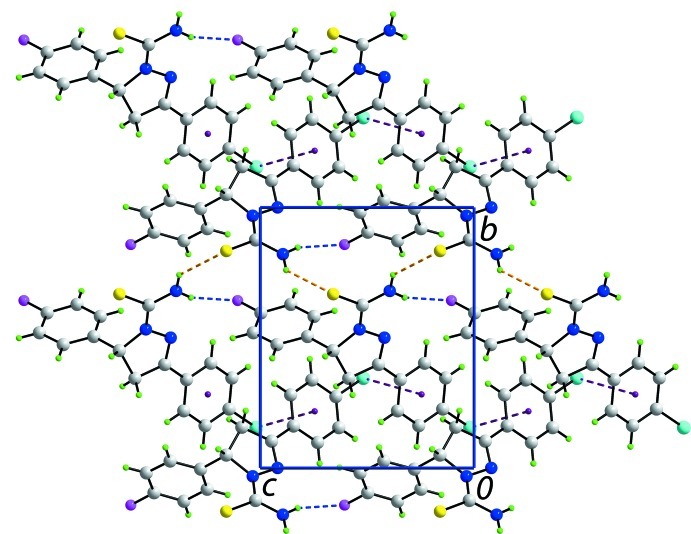
A view of the supramolecular layer in the bc plane in (I) mediated by N—H···S, N—H···F and Cl···π interactions, shown as orange, blue and purple dashed lines, respectively.

**Fig. 3.**
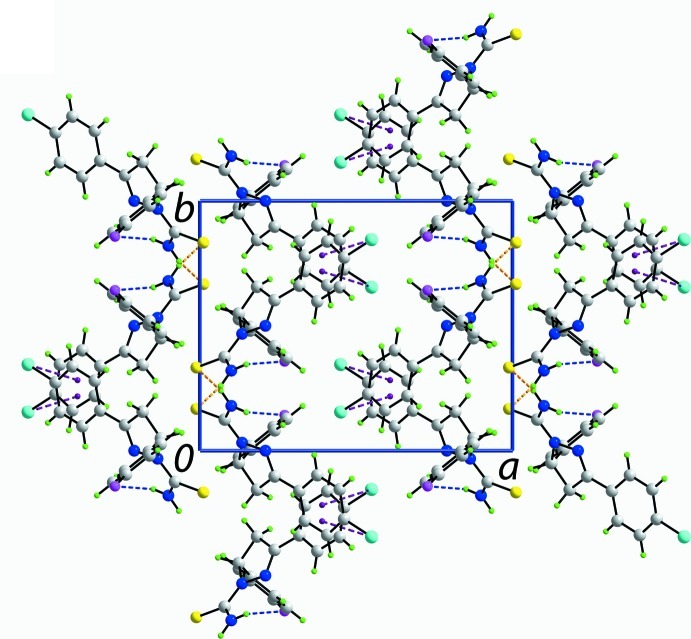
A view of the crystal packing in projection down the c axis. The N—H···S, N—H···F and Cl···π interactions are shown as orange, blue and purple dashed lines, respectively.

## supplementary crystallographic information

### Comment

Pyrazolin-1-ylthiazole derivatives are known to exhibit biological potential (Abdel-Wahab *et al.*, 2012; Abdel-Wahab *et al.*, 2009; Chimenti *et al.*, 2010) and motivated the investigation of the title compound, (I).

The central pyrazolyl ring in (I), Fig. 1, adopts an envelope conformation with the methine-C9 atom being the flap atom. The amine group is orientated towards the ring-N2 atom, forming a hydrogen bond, Table 1, assisted by the near co-planar relationship between the thioamide group and the pyrazolyl ring with the N1—N2—C16—N3 torsion angle being -0.7 (2)°. Both the chloro- and fluoro-benzene rings are twisted out of the least-squares plane through the five-membered ring, forming dihedral angles of 15.12 (11) and 80.55 (10)°, respectively. Quite similar conformations have been observed in related structures bearing two six-membered rings (Chantrapromma *et al.*, 2012; Abdel-Wahab *et al.*, 2013).

In the crystal packing, the amine-H31 atom participating in the intramolecular N—H···N hydrogen bond also forms a hydrogen bond to the F1 atom, Table 1. This interaction along with an N—H···S hydrogen bond leads to a supramolecular chain along the *c* axis, Table 1. Chains are connected into a layer in the *bc* plane by Cl···π interactions, Fig. 2 and Table 1. Layers stack along the *a* axis without specific interactions between them, Fig. 3.

### Experimental

To a suspension of (*E*)-1-(4-chlorophenyl)-3-(4-fluorophenyl)prop-2-en-1-one (1 mmol, 0.26 g) and sodium hydroxide (2.5 mmol, 1.0 g) in ethanol (20 ml), thiosemicarbazide (1.2 mmol, 0.11 g) was added. The mixture was refluxed for 12 h, then left to cool. The solid product was filtered off, washed with ethanol and dried. Recrystallization was by slow evaporation of its DMF solution.

### Refinement

Nitrogen- and carbon-bound H-atoms were placed in calculated positions (N—H = 0.88 Å, and C—H 0.93 to 0.98 Å) and were included in the refinement in the riding model approximation, with *U*_iso_(H) = 1.2–1.5*U*_equiv_(N,*C*).

### Crystal data

**Table:**

C_16_H_13_ClFN_3_S	*F*(000) = 688
*M**_r_* = 333.80	*D*_x_ = 1.464 Mg m^−^^3^
Monoclinic, *P*2_1_/*c*	Mo *K*α radiation, λ = 0.71073 Å
Hall symbol: -P 2ybc	Cell parameters from 2717 reflections
*a* = 14.5402 (9) Å	θ = 2.9–27.5°
*b* = 11.2700 (8) Å	µ = 0.40 mm^−^^1^
*c* = 9.5169 (6) Å	*T* = 295 K
β = 103.850 (6)°	Prism, colourless
*V* = 1514.17 (17) Å^3^	0.40 × 0.30 × 0.20 mm
*Z* = 4	

### Data collection

**Table:**

Agilent SuperNova Dual diffractometer with an Atlas detector	3478 independent reflections
Radiation source: SuperNova (Mo) X-ray Source	2570 reflections with *I* > 2σ(*I*)
Mirror monochromator	*R*_int_ = 0.031
Detector resolution: 10.4041 pixels mm^-1^	θ_max_ = 27.5°, θ_min_ = 2.9°
ω scan	*h* = −15→18
Absorption correction: multi-scan (*CrysAlis PRO*; Agilent, 2011)	*k* = −14→11
*T*_min_ = 0.898, *T*_max_ = 1.000	*l* = −12→12
10191 measured reflections	

### Refinement

**Table:**

Refinement on *F*^2^	Primary atom site location: structure-invariant direct methods
Least-squares matrix: full	Secondary atom site location: difference Fourier map
*R*[*F*^2^ > 2σ(*F*^2^)] = 0.041	Hydrogen site location: inferred from neighbouring sites
*wR*(*F*^2^) = 0.111	H-atom parameters constrained
*S* = 1.01	*w* = 1/[σ^2^(*F*_o_^2^) + (0.0432*P*)^2^ + 0.4541*P*] where *P* = (*F*_o_^2^ + 2*F*_c_^2^)/3
3478 reflections	(Δ/σ)_max_ = 0.001
199 parameters	Δρ_max_ = 0.19 e Å^−^^3^
0 restraints	Δρ_min_ = −0.28 e Å^−^^3^

### Special details

**Table:**

Geometry. All e.s.d.'s (except the e.s.d. in the dihedral angle between two l.s. planes) are estimated using the full covariance matrix. The cell e.s.d.'s are taken into account individually in the estimation of e.s.d.'s in distances, angles and torsion angles; correlations between e.s.d.'s in cell parameters are only used when they are defined by crystal symmetry. An approximate (isotropic) treatment of cell e.s.d.'s is used for estimating e.s.d.'s involving l.s. planes.
Refinement. Refinement of *F*^2^ against ALL reflections. The weighted *R*-factor *wR* and goodness of fit *S* are based on *F*^2^, conventional *R*-factors *R* are based on *F*, with *F* set to zero for negative *F*^2^. The threshold expression of *F*^2^ > σ(*F*^2^) is used only for calculating *R*-factors(gt) *etc*. and is not relevant to the choice of reflections for refinement. *R*-factors based on *F*^2^ are statistically about twice as large as those based on *F*, and *R*- factors based on ALL data will be even larger.

### Fractional atomic coordinates and isotropic or equivalent isotropic displacement parameters (Å^2^)

**Table:**

	*x*	*y*	*z*	*U*_iso_*/*U*_eq_	
Cl1	0.54931 (5)	0.65490 (7)	0.48095 (8)	0.0811 (3)	
S1	−0.01110 (4)	1.16708 (5)	−0.15948 (5)	0.04527 (17)	
F1	0.27125 (11)	1.14248 (13)	−0.60633 (13)	0.0658 (4)	
N1	0.20984 (11)	1.00001 (15)	0.08010 (15)	0.0379 (4)	
N2	0.13525 (11)	1.03202 (14)	−0.03592 (15)	0.0368 (4)	
N3	0.10065 (13)	1.17944 (16)	0.10514 (17)	0.0463 (4)	
H31	0.1483	1.1542	0.1741	0.056*	
H32	0.0669	1.2407	0.1207	0.056*	
C1	0.31834 (13)	0.83907 (18)	0.1544 (2)	0.0381 (4)	
C2	0.37595 (15)	0.9020 (2)	0.2678 (2)	0.0498 (5)	
H2	0.3661	0.9829	0.2772	0.060*	
C3	0.44754 (16)	0.8454 (2)	0.3665 (2)	0.0559 (6)	
H3	0.4860	0.8879	0.4419	0.067*	
C4	0.46163 (14)	0.7261 (2)	0.3529 (2)	0.0505 (6)	
C5	0.40755 (15)	0.6624 (2)	0.2408 (3)	0.0533 (6)	
H5	0.4187	0.5819	0.2314	0.064*	
C6	0.33602 (14)	0.7194 (2)	0.1414 (2)	0.0476 (5)	
H6	0.2993	0.6766	0.0647	0.057*	
C7	0.23925 (13)	0.89711 (18)	0.05223 (19)	0.0366 (4)	
C8	0.18302 (14)	0.84371 (17)	−0.08689 (19)	0.0394 (4)	
H8A	0.1424	0.7802	−0.0687	0.047*	
H8B	0.2242	0.8136	−0.1452	0.047*	
C9	0.12464 (13)	0.95057 (17)	−0.15998 (18)	0.0365 (4)	
H9	0.0581	0.9282	−0.1966	0.044*	
C10	0.16342 (12)	1.00448 (17)	−0.27969 (17)	0.0330 (4)	
C11	0.23938 (13)	1.08168 (18)	−0.24895 (19)	0.0403 (5)	
H11	0.2656	1.1032	−0.1534	0.048*	
C12	0.27723 (14)	1.12774 (19)	−0.3580 (2)	0.0442 (5)	
H12	0.3288	1.1791	−0.3370	0.053*	
C13	0.23618 (15)	1.09503 (19)	−0.4978 (2)	0.0430 (5)	
C14	0.16111 (15)	1.01890 (19)	−0.53370 (19)	0.0463 (5)	
H14	0.1350	0.9983	−0.6296	0.056*	
C15	0.12497 (14)	0.97331 (18)	−0.42252 (19)	0.0400 (4)	
H15	0.0741	0.9210	−0.4442	0.048*	
C16	0.07946 (13)	1.12480 (17)	−0.02300 (19)	0.0352 (4)	

### Atomic displacement parameters (Å^2^)

**Table:**

	*U*^11^	*U*^22^	*U*^33^	*U*^12^	*U*^13^	*U*^23^
Cl1	0.0556 (4)	0.0764 (5)	0.0967 (5)	0.0061 (3)	−0.0106 (3)	0.0318 (4)
S1	0.0410 (3)	0.0485 (4)	0.0446 (3)	0.0047 (2)	0.0067 (2)	0.0045 (2)
F1	0.0922 (10)	0.0679 (10)	0.0444 (7)	−0.0139 (8)	0.0304 (7)	0.0066 (6)
N1	0.0384 (8)	0.0440 (10)	0.0306 (7)	0.0025 (7)	0.0068 (6)	0.0007 (7)
N2	0.0406 (8)	0.0402 (10)	0.0295 (7)	0.0046 (7)	0.0080 (6)	−0.0024 (6)
N3	0.0537 (10)	0.0438 (10)	0.0409 (9)	0.0073 (8)	0.0106 (7)	−0.0069 (7)
C1	0.0368 (10)	0.0409 (12)	0.0380 (9)	0.0007 (8)	0.0116 (7)	0.0033 (8)
C2	0.0544 (13)	0.0427 (13)	0.0485 (12)	0.0034 (10)	0.0045 (9)	−0.0004 (9)
C3	0.0520 (13)	0.0587 (16)	0.0488 (12)	−0.0014 (11)	−0.0041 (10)	0.0004 (10)
C4	0.0349 (10)	0.0542 (15)	0.0606 (13)	0.0006 (10)	0.0078 (9)	0.0182 (11)
C5	0.0412 (11)	0.0397 (13)	0.0768 (15)	0.0024 (10)	0.0094 (10)	0.0066 (11)
C6	0.0400 (11)	0.0449 (13)	0.0565 (12)	−0.0026 (9)	0.0091 (9)	−0.0029 (10)
C7	0.0360 (10)	0.0397 (12)	0.0361 (9)	−0.0015 (8)	0.0129 (7)	0.0007 (8)
C8	0.0462 (11)	0.0372 (11)	0.0355 (9)	−0.0004 (9)	0.0111 (8)	−0.0002 (8)
C9	0.0364 (9)	0.0401 (11)	0.0324 (9)	−0.0022 (8)	0.0074 (7)	−0.0048 (8)
C10	0.0329 (9)	0.0343 (10)	0.0307 (8)	0.0027 (8)	0.0056 (7)	−0.0015 (7)
C11	0.0412 (10)	0.0458 (12)	0.0313 (9)	−0.0044 (9)	0.0033 (7)	−0.0028 (8)
C12	0.0431 (11)	0.0462 (13)	0.0437 (11)	−0.0074 (9)	0.0114 (8)	−0.0010 (9)
C13	0.0575 (12)	0.0397 (12)	0.0354 (10)	0.0030 (10)	0.0181 (9)	0.0038 (8)
C14	0.0605 (13)	0.0466 (13)	0.0289 (9)	−0.0014 (10)	0.0053 (8)	−0.0020 (8)
C15	0.0440 (11)	0.0380 (11)	0.0350 (9)	−0.0046 (9)	0.0037 (8)	−0.0030 (8)
C16	0.0378 (9)	0.0351 (11)	0.0358 (9)	−0.0042 (8)	0.0148 (7)	0.0024 (8)

### Geometric parameters (Å, º)

**Table:**

Cl1—C4	1.735 (2)	C5—H5	0.9300
S1—C16	1.6821 (19)	C6—H6	0.9300
F1—C13	1.365 (2)	C7—C8	1.505 (2)
N1—C7	1.285 (2)	C8—C9	1.540 (3)
N1—N2	1.397 (2)	C8—H8A	0.9700
N2—C16	1.347 (2)	C8—H8B	0.9700
N2—C9	1.474 (2)	C9—C10	1.514 (3)
N3—C16	1.335 (2)	C9—H9	0.9800
N3—H31	0.8800	C10—C11	1.381 (3)
N3—H32	0.8800	C10—C15	1.386 (2)
C1—C6	1.384 (3)	C11—C12	1.386 (3)
C1—C2	1.392 (3)	C11—H11	0.9300
C1—C7	1.470 (3)	C12—C13	1.373 (3)
C2—C3	1.380 (3)	C12—H12	0.9300
C2—H2	0.9300	C13—C14	1.366 (3)
C3—C4	1.371 (3)	C14—C15	1.388 (3)
C3—H3	0.9300	C14—H14	0.9300
C4—C5	1.367 (3)	C15—H15	0.9300
C5—C6	1.385 (3)		
			
C7—N1—N2	107.67 (15)	C7—C8—H8B	111.4
C16—N2—N1	119.92 (15)	C9—C8—H8B	111.4
C16—N2—C9	127.35 (15)	H8A—C8—H8B	109.2
N1—N2—C9	112.63 (14)	N2—C9—C10	111.49 (15)
C16—N3—H31	120.0	N2—C9—C8	100.63 (13)
C16—N3—H32	120.0	C10—C9—C8	112.91 (15)
H31—N3—H32	120.0	N2—C9—H9	110.5
C6—C1—C2	118.41 (18)	C10—C9—H9	110.5
C6—C1—C7	120.48 (18)	C8—C9—H9	110.5
C2—C1—C7	121.10 (19)	C11—C10—C15	118.82 (17)
C3—C2—C1	120.5 (2)	C11—C10—C9	121.08 (15)
C3—C2—H2	119.7	C15—C10—C9	120.06 (17)
C1—C2—H2	119.7	C10—C11—C12	121.19 (17)
C4—C3—C2	119.7 (2)	C10—C11—H11	119.4
C4—C3—H3	120.2	C12—C11—H11	119.4
C2—C3—H3	120.2	C13—C12—C11	117.83 (19)
C3—C4—C5	121.16 (19)	C13—C12—H12	121.1
C3—C4—Cl1	119.23 (17)	C11—C12—H12	121.1
C5—C4—Cl1	119.61 (19)	C14—C13—F1	118.55 (17)
C4—C5—C6	119.1 (2)	C14—C13—C12	123.16 (18)
C4—C5—H5	120.4	F1—C13—C12	118.28 (19)
C6—C5—H5	120.4	C13—C14—C15	117.89 (17)
C5—C6—C1	121.1 (2)	C13—C14—H14	121.1
C5—C6—H6	119.5	C15—C14—H14	121.1
C1—C6—H6	119.5	C10—C15—C14	121.10 (18)
N1—C7—C1	120.74 (17)	C10—C15—H15	119.4
N1—C7—C8	113.88 (16)	C14—C15—H15	119.4
C1—C7—C8	125.33 (18)	N3—C16—N2	115.42 (16)
C7—C8—C9	102.08 (15)	N3—C16—S1	122.79 (16)
C7—C8—H8A	111.4	N2—C16—S1	121.78 (14)
C9—C8—H8A	111.4		
			
C7—N1—N2—C16	167.15 (16)	C16—N2—C9—C8	−159.69 (18)
C7—N1—N2—C9	−9.4 (2)	N1—N2—C9—C8	16.59 (19)
C6—C1—C2—C3	−1.4 (3)	C7—C8—C9—N2	−16.22 (18)
C7—C1—C2—C3	177.32 (19)	C7—C8—C9—C10	102.72 (17)
C1—C2—C3—C4	−0.3 (3)	N2—C9—C10—C11	31.9 (2)
C2—C3—C4—C5	1.8 (4)	C8—C9—C10—C11	−80.5 (2)
C2—C3—C4—Cl1	−177.91 (17)	N2—C9—C10—C15	−150.42 (17)
C3—C4—C5—C6	−1.4 (4)	C8—C9—C10—C15	97.1 (2)
Cl1—C4—C5—C6	178.23 (17)	C15—C10—C11—C12	−0.1 (3)
C4—C5—C6—C1	−0.3 (3)	C9—C10—C11—C12	177.60 (18)
C2—C1—C6—C5	1.7 (3)	C10—C11—C12—C13	0.6 (3)
C7—C1—C6—C5	−177.00 (19)	C11—C12—C13—C14	−0.7 (3)
N2—N1—C7—C1	179.67 (16)	C11—C12—C13—F1	178.21 (18)
N2—N1—C7—C8	−2.9 (2)	F1—C13—C14—C15	−178.68 (19)
C6—C1—C7—N1	165.27 (18)	C12—C13—C14—C15	0.2 (3)
C2—C1—C7—N1	−13.4 (3)	C11—C10—C15—C14	−0.4 (3)
C6—C1—C7—C8	−11.9 (3)	C9—C10—C15—C14	−178.12 (18)
C2—C1—C7—C8	169.41 (19)	C13—C14—C15—C10	0.3 (3)
N1—C7—C8—C9	12.9 (2)	N1—N2—C16—N3	−0.7 (2)
C1—C7—C8—C9	−169.76 (17)	C9—N2—C16—N3	175.38 (17)
C16—N2—C9—C10	80.3 (2)	N1—N2—C16—S1	179.89 (13)
N1—N2—C9—C10	−103.38 (17)	C9—N2—C16—S1	−4.1 (3)

### Hydrogen-bond geometry (Å, º)

Cg1 is the centroid of the C1–C6 ring.

**Table:**

*D*—H···*A*	*D*—H	H···*A*	*D*···*A*	*D*—H···*A*
N3—H31···N1	0.88	2.24	2.617 (2)	106
N3—H31···F1^i^	0.88	2.41	3.257 (2)	163
N3—H32···S1^ii^	0.88	2.81	3.5203 (19)	139
C4—Cl1···*Cg*1^iii^	1.74 (1)	3.92 (1)	4.183 (2)	86 (1)

Symmetry codes: (i) *x*, *y*, *z*+1; (ii) *x*, −*y*+5/2, *z*+1/2; (iii) *x*, −*y*+1/2, *z*−1/2.

## References

[bb1] Abdel-Wahab, B. F., Abdel-Aziz, H. A. & Ahmed, E. M. (2009). *Eur. J. Med. Chem.* **44**, 2632–2635. 10.1016/j.ejmech.2008.09.029 18995932

[bb2] Abdel-Wahab, B. F., Abdel-Latif, E., Mohamed, H. A. & Awad, G. E. A. (2012). *Eur. J. Med. Chem.* **52**, 263–268. 10.1016/j.ejmech.2012.03.023 22480494

[bb3] Abdel-Wahab, B. F., Mohamed, H. A., Khidre, R. E., Ng, S. W. & Tiekink, E. R. T. (2013). Acta Cryst. E**69**, o386. 10.1107/S1600536813004194 PMC3588501 23476571

[bb4] Agilent (2011). *CrysAlis PRO* Agilent Technologies, Yarnton, England.

[bb5] Brandenburg, K. (2006). *DIAMOND* Crystal Impact GbR, Bonn, Germany.

[bb6] Chantrapromma, S., Nonthason, P., Suwunwong, T. & Fun, H.-K. (2012). *Acta Cryst.* E**68**, o830–o831. 10.1107/S1600536812006642 PMC3297890 22412693

[bb7] Chimenti, F., Carradori, S., Secci, D., Bolasco, A., Bizzarri, B., Chimenti, P., Granese, A., Yáñez, M. & Orallo, F. (2010). *Eur. J. Med. Chem.* **45**, 800–804. 10.1016/j.ejmech.2009.11.003 19926363

[bb8] Farrugia, L. J. (2012). *J. Appl. Cryst.* **45**, 849–854.

[bb9] Sheldrick, G. M. (2008). *Acta Cryst.* A**64**, 112–122. 10.1107/S0108767307043930 18156677

[bb10] Westrip, S. P. (2010). *J. Appl. Cryst.* **43**, 920–925.

